# Time, cause of early neonatal death, and its predictors among neonates admitted to neonatal intensive care units at Bahir Dar City public hospitals, northwest Ethiopia: a prospective follow-up study

**DOI:** 10.3389/fped.2024.1335858

**Published:** 2024-06-11

**Authors:** Abraham Dessie Gessesse, Minyichil Birhanu Belete, Fikir Tadesse

**Affiliations:** ^1^ Department of Nursing, College of Medicine and Health Science, Dilla University, Dilla, Ethiopia; ^2^ Department of Pediatrics and Child Health Nursing, College of Medicine and Health Sciences, Bahir Dar University, Bahir Dar, Ethiopia

**Keywords:** death, incidence, newborn, cause, Ethiopia

## Abstract

**Background:**

Globally, 75% of neonatal deaths occur during the first weeks of life and more than 43% of deaths are covered by sub-Saharan Africa. Health-related policymakers and decision-makers need to use evidence-based treatments to reduce the time to early neonatal death and associated predictors. However, there are limited studies on median survival time, cause, incidence, and predictors in the study area as well as the country. Therefore, the aim of the present study was to assess time, the cause of early neonatal death, and its predictors among neonates admitted to neonatal intensive care units at Bahir Dar City public hospitals in northwest Ethiopia.

**Methods:**

An institution-based prospective follow-up study design was conducted among 387 early neonates selected by systematic sampling between 22 February and 22 April 2023. Statistical software, Epi Data version 4.6 and Stata version 14, was used for entry and analysis, respectively. Proportional hazard assumption and model fitness were checked by the Schoenfeld residual test and the Cox-Snell residual test, respectively. Descriptive statistics, the Kaplan–Meier curve, and the life table were used to describe variables. The Cox regression analysis model was fitted to identify the predictors of early neonatal death.

**Result:**

During the follow-up time, 59 (15.25%) early neonates died, with an incidence of 31.79 per 1,000 early neonate days [95% confidence interval (CI): 0.024–0.041]. The leading causes of early neonatal death were prematurity complications, asphyxia, sepsis, meconium aspiration syndrome, and necrotizing enterocolitis. The mean survival time was 2.72 days. Being born from a multigravida mother [adjusted hazard ratio (AHR) 4.34; 95% CI: 1.63–11.55], a grand multigravida mother (AHR 3.50; 95% CI: 1.12–10.95), respiratory distress syndrome (AHR 2.60; 95% CI: 1.03–6.58), birth asphyxia (AHR 7.51; 95% CI: 2.30–24.51), a small gestational age (AHR 2.05; 95% CI: 1.08–4.92), and being unable to exclusively breastfeed (AHR 3.46; 95% CI: 1.52–7.88) were significantly associated predictors for time to early neonatal death.

**Conclusion and recommendations:**

The incidence of early neonatal death was high, and the mean survival time was 2.72 days. Gravidity, respiratory distress syndrome, birth asphyxia, and being unable to exclusively breastfeed were identified as predictors of early neonatal death. Therefore, future research will consist of long-term prospective follow-up studies at a multicenter, nationwide level.

## Introduction

Early neonatal age is defined as the first 7 days of birth after delivery, which is generally regarded as the most vulnerable time in a child's life (1, 2). It is a time when all physiological changes are very rapid and the infant may experience crucial events, such as feeding patterns being formed, parental bonding beginning, the risk of illnesses that may grow more serious being increased, and many birth or congenital problems being first detected (3). The term “early neonatal death,” which refers to a neonatal death between birth and 7 full days after birth, contributes considerably to total neonatal death (4, 5).

Even though early neonates are not a disease, the majority of deaths occur within 7 days of life, since they face difficulties in the external environment, which makes them the most vulnerable and highest risk of dying soon after birth (6, 7). Early neonates are the most susceptible age group of neonates and remain a public health concern (8). Early neonates have the largest risk of death worldwide, which varies by region, and have a 56 times higher chance than countries with low death rates, including sub-Saharan countries (9). In 2020, each day there were approximately 6,500 early neonatal deaths (ENDs) globally, 25% of which occurred on the first day of life and 75% in the first week (9). In the 2022 global report, sub-Saharan Africa (SSA) has the highest neonatal death rate (27 deaths per 1,000 live births), accounting for 43% of all neonatal deaths globally, followed by central and southern Asia (23 deaths per 1,000 live births), accounting for 36% of all neonatal deaths globally.

The distribution of neonatal deaths varies across regions, based on income, educational status, and age. A total of 10 countries account for 75% of global neonatal deaths: India (4.9%), Nigeria (2.71%), Pakistan (2.44%), Ethiopia (0.97%), the Democratic Republic of the Congo (0.96%), China (0.56%), Indonesia (0.56%), Bangladesh (0.51%), Afghanistan (0.43%), and the United Republic of Tanzania (0.43%) (10).

The trend between 2017 and 2018 was that 1 in 36 neonates in SSA died, compared to 1 in 333 in high-income countries around the world; therefore, with this trend between 2017 and 2030, 30 million neonates will die in 2030 (11). Even though the global neonatal death rate has decreased from 37 deaths per 1,000 live births in 1990 to 18 deaths per 1,000 live births in 2018, the likelihood that an infant will survive varies greatly depending on the region and country in which they are born (12). About 40% of sub-Saharan countries did not experience a significant decrease in neonatal deaths between 1990 and 2018 compared to other higher-income countries (11, 13, 14).

Ethiopia has the second-highest rate of neonatal deaths in SSA and is ranked fourth worldwide (10, 15). According to the Mini Ethiopian Demographic Health Survey (DHS), new neonatal deaths increased significantly, from 0.029% in 2016 to 0.033% in 2019, even though there were regional variations, such as the Amhara region, which reports the second largest number of neonatal deaths, accounting for 0.046% of deaths next to Benishangul Gumuz, which has 0.055% of deaths (16).

Prematurity and congenital abnormalities are the main leading causes of ENDs in high-income countries. Furthermore, only recently have sudden, unexpected early neonatal deaths been recognized as important and frequently avoidable causes of death. In addition, preterm and perinatal-related factors such as hypoxia and infections are the main causes of END in Africa, Southeast Asia, and Latin America (17). According to the WHO in 2022, preterm delivery, hypoxia, infection, and birth defects were the primary causes of END. Preterm-related complications were one of the leading causes of death for 30%–35% of ENDs (10, 18). In low-income countries, including Ethiopia, the causes of END were neonatal infections, low weight, neonatal distress syndrome (RDS), low appearance, pulse, grimace, activities, respiration (APGAR) scores, preterm delivery, not receiving ANC follow-up, delaying the start of breastfeeding, birth defects, hypoxia, small for gestational age (SGA), home birth, insufficiencies in access to healthcare, birth spacing, and length of hospital stay (19–24).

As a result of the early neonatal age group having the greatest death and morbidity rates ever, it has effects on socioeconomic consequences, psychological burden, emotional trauma, parents’ loss of potential support in their older age, and negatively affects the availability of household labor as well as having economic effects on family life, community health, and the entire nation (25). Ethiopia also embraces several initiatives, such as the extension of Sustainable Development Goal 3 Target 2 and the continuation of the Millennium Development Plan's incomplete agenda, to reduce neonatal deaths by at least 0.012% of total deaths by 2030 (26). However, more than 75% of ENDs will be preventable and treatable at an affordable cost if people are aware of hazards, know predictors of time to death, avail adequate scientific data for ENDs in each health institution, concentrate studies on the first weeks of life, and give much attention to this age group. However, there is limited evidence to show incidence, median survival time, and predictors of death in different parts of Ethiopia, specifically the study area. Therefore, the aims of this study were to assess the time, causes of early neonatal death, and its predictors among neonates admitted to neonatal intensive care units at Bahir Dar City public hospitals in northwest Ethiopia.

## Methods

### Study area and period

A study was conducted at Bahir Dar City public hospitals. Bahir Dar City is located in the northwest of Ethiopia, which is 565 km from Addis Ababa, the capital of Ethiopia. It is the headquarters of Amhara regional state, which is bounded by the Blue Nile River and Lake Tana. Currently, the city has three governmental hospitals. Among these hospitals are Felege Hiwot Comprehensive Specialized Hospital (FHCSH), Tibebe Ghion Specialized Hospital (TGSH), and Addis Alem Primary Hospital (AAPH). Felege Hiwot Comprehensive Specialized Hospital provides services for 3,500 admitted neonates annually; among these, the average monthly early neonatal admission was 180. TGSH is another public hospital in the city. Its neonatal intensive care unit (NICU) has separate rooms for preterm and term neonates. There were 160 early neonates admitted to NICU wards on average per month. Another public hospital in the city is AAPH. Its NICU receives an average of 45 early neonates every month. The study was carried out between 22 February and 22 April 2023.

### Study design, population, and eligibility criteria

An institutional-based prospective follow-up study design was employed. The source populations are early neonates who were admitted to the NICU at Bahir Dar City public hospitals in the northwest of Ethiopia during the study period.

Study samples are all selected early neonates who were admitted to the NICU at Bahir Dar City public hospitals between 22 February and 22 April 2023. The early neonate's carer, who was unable to communicate due to illness, and the early neonates who were referred after being admitted to public hospitals in Bahir Dar City were excluded from the study.

### Sample size and sampling methods

The sample size was determined using Stata software version 14 by major predictors in the previous study (20, 21). Among those predictors, birth asphyxia was an independent predictor that gave a maximum sample size of 351 (Supplementary Table S1). After adding a 10% loss follow-up, a 95% confidence interval (CI), and an 80% power, the final sample size was 387. This sample was proportionally distributed to the three hospitals using the average 6-month preceding data analysis. Based on this data analysis, the mean number of early neonatal admissions per 2 months and *k* value of 2 was 90 for AAPH, 360 for FHCSH, and 320 for TGSH, using systematic sampling from the three hospitals with the first neonate selected through lottery methods, and then using the proportional allocation formula there were 45, 161, and 181 recruits from AAPH, TGSH, and FHCSH, respectively (Supplementary Figure S1).

### Study variables

The outcome variable were time and cause of early neonatal deaths, coded as (1 = event and 0 = censored). Independent variables included the following sociodemographic factors: maternal age; age of neonate; sex of neonate; mother’s level of education (cannot read and write, can read and write only, primary learning, secondary learning, college and above); mother’s occupation (governmental employee, private, merchant, farmer, housewife); and mother’s residence (urban or rural). Maternal medical and obstetric-related predictors were as follows: parity (primi para, multipara, grand-multi para); gravidity (primigravida, multigravida, grand multigravida); time of birth interval (no previous birth, birth interval <2 years, birth interval ≥2 years); time of rupture of membrane (<12 and ≥12 h); duration of labor (<18/≥18 h); onset of labor [spontaneous, induce, and elective cesarean delivery (C/S)]; history of abortion (yes/no); history of preterm (yes/no); current pregnancy complication (yes/no); complications during labor (yes/no); maternal chronic disease (yes/no); and type of pregnancy (singleton or multiple) (yes/no).

Neonatal-related admission predictors included the following: fetal congenital anomaly (yes/no); birth weight (<5,000 g/≥2,500 g); prematurity (yes/no); asphyxia (yes/no); jaundice (yes/no); size of gestational age (AGA, SGA, and LGA); gestational age (term/preterm); hypothermic (yes/no); birth order (first, second, third, fourth, and above); APGAR score at 5 min (<7/≥7); sepsis (yes/no); RDS (yes/no); and meconium aspiration syndrome (MAS) (yes/no). Treatment and health service-related predictors were as follows: trained NICU staff (yes/no); place of delivery (health facility/home); kangaroo mother care (yes/no); CPAP (yes/no); resuscitation (yes/no); number of ANC (1–3, ≥4); length of stay (<24/≥24 h); PNC (yes/no); birth attendant (skilled health professional/family or TBA); time of breastfeeding (<1/>1 h); EBF (yes/no); and mode of delivery (SVD/C/S) (Supplementary Figure S2).

### Operational definitions

The following are definitions of terms used in this study:

Early neonate, a neonate in the first week of life after delivery (1);

Early neonatal death, the death of a neonate during the follow-up (4);

Time to early neonatal death is the time in days from admission to the event within 7 days;

• The event is an early neonatal death;
• “Censored” refers to early neonates who were alive at the end of follow-up, lost to follow-up, referred, or discharged due to improvement;
• Exposure time is the time starting from the time of admission;
• The follow-up period is a period from admission to the time of death or censored until 7 days;
• Carers might be the neonate's mother, father, or family member;
• Early-onset neonatal sepsis refers to sepsis in a neonate at or before 72 h of life (some experts use 7 days) (27);
• Late-onset neonatal sepsis is defined as sepsis occurring at or after 72 h of life (27).
• RDS is a condition characterized by grunting while breathing, rapid or shallow breathing, and flaring of nostrils (28);
• Perinatal asphyxia has an APGAR score that remained at less than 7 (at 5 min after birth) and evidence of acute hypoxic compromise with acidemia (29);
• MAS is a clinical condition characterized by respiratory failure occurring in neonates born through meconium-stained amniotic fluid whose symptoms cannot be otherwise explained and with typical radiological characteristics (30);
• Neonatal hypothermia is a progressive reduction in the axillary temperature of the newborn (temperature <36.50°C) (31);
• Necrotizing enterocolitis (NEC) is an acute inflammatory disease of the intestine which primarily affects preterm infants and is a leading cause of morbidity and mortality in the neonatal intensive care unit (32).

### Data collection tools and procedures

The data collection tools were interview questionaries, observation, and follow-up checklists, which were used and adapted from pertinent literature (19–22, 23, 33–34) and WHO standard verbal autopsy (35). The carer, chart, and early neonates were interviewed, observed, and followed using those tools, respectively. The interview questionnaire contains the sociodemographic predictors of the mother. The observation checklist contains the mother's medical and obstetric predictors, neonatal sociodemographic information, and date of admission. The follow-up checklist included neonatal treatment, health service-related predictors, and neonatal outcomes. The data were then collected by looking over the maternal chart, doing a maternal interview, following the neonatal chart, and observing the neonates. At each of the three sites, a total of three BSc nurses gathered the data and one supervisor monitored daily activities. The collection of data was started on the day of admission up to 7 days of age. The first day of admission was taken as the starting time of the follow-up, while the censored and dead neonates were considered the ends of the follow-up. The time to END was calculated in days using the time interval between the time of admission and death. Seven completed days was the maximum number of follow-up days. Even though the data collectors were assigned by the volunteers of each respective NICU staff, they visited the neonates twice a day, and handover may have occurred on days off or during work hours. During the follow-up, if a death occurred, the data collector must have taken note of the date and reviewed the death summary to ascertain the cause of death.

### Data processing and analysis

Data were entered via Epi Data version 4.6 and then checked, cleaned, edited, coded, and exported to Stata version 14 for analysis. Descriptive statistics were shown using mean, median, standard deviation, frequency, table, graph, and percentage, depending on the nature of the variable. The incidence density rate was also calculated for the entire study period. The proportional hazard assumption was checked using the Schoenfeld residual test. The test resulted in a *p*-value for each covariate and the entire covariate simultaneously greater than 0.05, indicating that there are no time-varying covariates (Supplementary Table S2). A Kaplan–Meier curve with the Cox equality test was used to compare the survival curve of covariates and the risk of dying (Supplementary Table S3). Multicollinearity was checked through the variance inflation factor. After that, ANC, parity, preterm, and place of delivery were omitted due to multicollinearity. The overall mean variance inflation factor was 3.32 (Supplementary Table S3). The model's fitness was checked using the Cox-Snell residuals test. The Cox-Snell residual line and jagged line roughly follow 45° lines; because of this, the entire Cox regression model fits the data (Supplementary Figure S3). A life table was used to estimate the median survival time and the cumulative probability of death. The time to early neonatal death was calculated in days using the time interval between the time of admission and death. Information about the number of deaths and censors was collected from the daily follow-up checklist. The survival time of the predictors was calculated in days from the time of admission until death. To find predictors of time to END, bivariable and multivariable Cox regression analyses were used. If the *p*-value in the bivariable Cox regression analysis is less than 0.25, it is statistically a candidate for multivariable Cox regression. Finally, an adjusted hazard ratio was used to summarize the association, and *p*-values <0.05 with a 95% CI were used to declare statistical significance.

### Data quality assurance

To ensure the quality of the data, numerous attempts were made, such as adapting the tool using pertinent literature and WHO verbal autopsy. This tool was reviewed before data collection began by research experts, an MSc neonatology nurse specialist research expert, and ultimately, research advisors. The investigator provided a 1-day training session about the specifics of the checklist's contents, the technique, the aim of the study, the time to record and follow, and ethical considerations at each hospital, for a total of 3 days. There were three BSc nurse data collectors and a supervisor (an MSc clinical midwife student). The supervisor was monitoring all data collectors’ activities and the consistency, completeness, and clarity of the data. The supervisor and investigator received input from data collectors on daily activities, and all site data collectors received a similar response. Pretesting was carried out with 5% of early neonates at Gondar Compressive Specialized Teaching Hospital, which has similar sociodemographic characteristics to the study area, before the actual data collection time. It was modified in light of the results of this testing, but these findings were not used in the final data analysis.

## Results

### Sociodemographic characteristics of the mother and the neonate

From a total of 803 admitted early neonates, a selected 387 early neonates were followed in the NICU of Bahir Dar City public hospitals between 22 February and 22 April 2023. The response rate was 100%. In total, 304 (78.55%) index mothers were aged 20–34 years, and 44 (74.58%) deaths were observed in this age category. The median age of the mothers was 28 years [interquartile range (IQR): 21–36.5]. In this study, 228 (58.91%) mothers lived in urban areas, and 55.93% of early neonatal deaths occurred in rural areas. In the current study, 227 (58.66%) neonates were male, and 50.85% of deaths were male compared to female neonates. In total, 281 (72.61%) early neonates at admission were aged <1 day; three-fourths of early neonatal deaths occurred in this age category, and the median age of the neonates at admission was 0.42 days (IQR: 0.02–3) (Table 1).

**Table 1:** Sociodemographic characteristics of early neonates and their index mothers among neonates admitted to the neonatal intensive care unit of Bahir Dar City public hospitals, northwest Ethiopia, 2023.

Variable	Categorical	Total, *n* (%)	Status of the neonate	
			Censored, *n* (%)	Death, *n* (%)
Age of the mother	15–19	13 (3.36)	11 (3.35)	2 (3.39)
	20–34	304 (78.55)	260 (79.27)	44 (74.58)
	35–49	70 (18.09)	57 (17.38)	13 (22.03)
Sex of the neonate	Male	227 (58.66)	197 (60.06)	30 (50.85)
	Female	160 (41.34)	131 (39.94)	29 (49.15)
Age of the neonate	<1 days	281 (72.61)	237 (72.26)	44 (74.58)
	2–7 days	106 (27.39)	91 (27.74)	15 (25.42)
Residency	Urban	228 (58.91)	202 (61.59)	26 (44.07)
	Rural	159 (41.09)	126 (38.41)	33 (55.93)
Education level	Cannot read and write	118 (30.49)	92 (30.07)	26 (44.07)
	Can read and write	40 (10.34)	33 (10.06)	7 (11.86)
	Primary learning	57 (14.73)	52 (15.86)	5 (1.52)
	Secondary learning	64 (16.54)	56 (17.07)	8 (13.59)
	College and above	108 (27.91)	95 (28.96)	13 (22.03)
Mother occupation	Government employee	58 (14.99)	51 (15.55)	7 (11.86)
	Marchant	22 (5.68)	19 (5.79)	3 (5.08)
	Private	58 (14.99)	50 (15.24)	8 (13.56)
	Farmer	78 (20.16)	56 (17.07)	22 (37.29)
	Housewife	171 (44.19)	146 (52.13)	19 (32.20)

### Maternal medical and obstetrics predictors

In this study, 190 (49.10%) mothers were multi para and 54.24% of early neonatal death were observed in this group. This finding indicates that 156 (40.31%) mothers were predominately primigravida, and 52.54% and 25.42% of early neonatal deaths were observed in multigravida and grand-multigravida mothers, respectively. In the present study, 50 early neonates died. Of the mothers who had labored less than 18 h, 327 (84.50%) index mothers had spontaneous onset of labor (Table 2).

**Table 2:** Maternal medical and obstetric characteristics of index mothers among early neonates admitted to the neonatal intensive care unit of Bahir Dar City public hospitals, northwest Ethiopia, 2023.

Variable	Categorical	Total, *n* (%)	Status of the neonate	
			Censored, *n* (%)	Death, *n* (%)
Parity	Primi para	165 (42.64)	145 (44.21)	20 (33.90)
	Multi para	190 (49.10)	158 (48.17)	32 (54.24)
	Grand multipara	32 (8.27)	25 (7.62)	7 (11.86)
Gravidity	Primi gravida	156 (40.31)	143 (43.60)	13 (22.03)
	Multigravida	149 (38.50)	118 (35.98)	31 (52.54)
	Grand-multi gravida	82 (21.19)	67 (11.43)	15 (25.42)
Type of pregnancy	Single	293 (75.71)	257 (78.35)	36 (61.02)
	Multiple	94 (24.29)	71 (21.65)	23 (38.98)
Time of PROM	<12 h	15 (34.88)	13 (34.21)	2 (40.00)
	≥12 h	28 (65.12)	25 (65.79)	3 (60.00)
Pregnancy-related complication	Yes	36 (9.30)	29 (8.84)	7 (11.86)
	No	351 (90.70)	299 (91.16)	52 (88.14)
Maternal chronic disease	Yes	18 (4.65)	17 (5.18)	1 (1.69)
	No	369 (95.35)	311 (94.82)	58 (98.31)
Time of birth interval	No previous birth	119 (30.74)	104 (31.71)	15 (25.42)
	Birth interval < 2 years	114 (29.46)	98 (29.88)	16 (27.12)
	birth interval ≥2 years	154 (39.79)	126 (38.41)	28 (47.46)
History of preterm	Yes	17 (3.39)	9 (2.74)	8 (13.56)
	No	370 (95.61)	319 (97.26)	51 (86.44)
Duration of labor	<18 h	337 (96.29)	287 (89.69)	50 (90.91)
	≥18 h	13 (3.710	8 (2.71)	5 (9.090)
Onset of labor	Spontaneous	327 (84.50)	277 (84.45)	50 (84.75)
	Induce	23 (5.94)	18 (5.49)	5 (8.47)
	Elective C/s	37 (9.56)	33 (10.06)	4 (6.78)
Complication during labor	Yes	112 (28.94)	98 (29.88)	14 (23.72)
	No	275 (71.06)	230 (70.12)	45 (76.27)
History of abortion	Yes	48 (12.40)	42 (12.80)	6 (10.17)
	No	339 (87.60)	28 (97.12)	53 (89.83)

Pregnancy-related complications (APH, pre-eclampsia, G-HTN, G-DM). Maternal chronic disease (DM, HTN, HIV/AIDS, HBV, HCV, CRVHD, anemia), Complication during labor (prolonged labor, fetal distress, rupture of uterine, cord prolapse, malpresentation, oligohydramnios).

### Early neonatal admission diagnosis-related characteristics

Of 387 followed early neonates, 172 (44.44%) were firstborn children; 24 (40.68%) deaths occurred in this group. In this follow-up study, the median gestational age was 37 weeks (IQR: 30.5–41). Of the early neonates, 243 (62.79%) were appropriate for gestational age, and 67.80% of early neonatal deaths were small for gestational age. According to our study, the median weight of the early neonates at admission was 2,445 g (IQR: 1,335–3,550) and 46 (77.97%) ENDs were observed in admissions with a weight less than 2,500 g. There were 163 (42.12%) premature early neonates, of which 43 (72.88%) died. Among 59 (15.25%) neonates with RDS and 55 (14.21%) neonates with birth asphyxia, 57.63% and 28.81% of deaths occurred in RDS and birth asphyxia, respectively (Table 3).

**Table 3:** Early neonatal-related characteristics among admitted neonates to the neonatal intensive care unit of Bahir Dar City public hospitals, northwest Ethiopia, 2023.

Variable	Categorical	Total, *n* (%)	Status of the neonates	
			Censored, *n* (%)	Death, *n* (%)
Birth order	First baby	172 (44.44)	148 (45.12)	24 (40.68)
	Second baby	93 (24.03)	78 (23.78)	15 (25.42)
	Third baby	48 (12.40)	43 (13.11)	5 (8.47)
	Fourth and above	74 (19.12)	58 (17.68)	15 (25.42)
Size of GA	Appropriate for GA	243 (62.79)	225 (68.60)	18 (30.51)
	Small for GA	120 (31.01)	80 (24.39)	40 (67.80)
	Large for GA	24 (6.20)	23 (7.01)	1 (1.69)
Birth weight	<2,500 gm	194 (50.13)	148 (45.12)	46 (77.97)
	≥2,500 gm	193 (49.87)	180 (54.88)	13 (22.03)
Sepsis	Yes	317 (81.91)	264 (80.49)	53 (89.83)
	No	70 (18.09)	64 (19.51)	6 (10.17
RDS	Yes	59 (15.25)	25 (7.62)	34 (57.63)
	No	328 (84.75)	303 (92.38)	25 (42.37)
Asphyxia	Yes	55 (14.21)	38 (11.59)	17 (28.81)
	No	332 (85.79)	290 (88.41)	42 (71.19)
MAS	Yes	37 (9.56)	31 (9.45)	6 (10.17)
	No	350 (90.44)	297 (90.55)	53 (89.83)
Hypothermia	Yes	247 (63.820	198 (60.37)	49 (83.05)
	No	140 (36.18)	130 (39.63)	10 (16.95)
NHB	Yes	35 (9.04)	32 (9.76)	3 (5.08)
	No	352 (90.96)	296 (90.24)	56 (17.07)
APGAR done	Yes	267 (68.99)	233 (71.04)	34 (57.63)
	No	120 (31.01)	95 (28.96)	25 (42.37)
5th min APGAR score	<7	33 (12.36)	23 (9.87)	10 (29.41)
	≥7	234 (87.64)	210 (90.13)	24 (70.59)
Congenital anomaly	Yes	19 (4.91)	17 (5.18)	2 (3.39)
	No	368 (95.09)	311 (94.82)	57 (96.61)
Prematurity	Yes	163 (42.12)	120 (36.59)	43 (72.88)
	No	224 (57.88)	208 (63.41)	16 (27.12)
Hypoglycemia	Yes	17 (4.39)	13 (3.96)	4 (6.78)
	No	370 (95.61)	315 (96.04)	55 (93.22)
Dehydration	Yes	39 (10.08)	34 (10.37)	5 (8.47)
	No	348 (89.92)	294 (89.63	54 (98.31)
Other^a^	Yes	32 (8.27)	26 (7.93)	6 (10.17)
	No	302 (91.73)	302(92.07)	53(89.83)

^a^ Others (polycythemia, Patau's syndrome, omphalocele, apnea, rupture mylomengocelle, subgalial hemorrhage).

### Treatment and health service-related predictor

Of 387 followed early neonates, 378 (97.67%) were delivered from mothers with ANC follow-up; among those mothers, 205 (52.97%) had more than four visits. In this study, 50.13% of the early neonates started initiation of breastfeeding within 1 h; however, 31 (52.54%) of them died. According to this study, the median length of stay was 6 days (IQR: 1–7). In total, 344 (88.89%) of the early neonates stayed more than 1 day, of which 39 (66.10%) died. Regarding the admitted early neonates, 269 (69.51%) initiated exclusive breastfeeding, and 37 (62.71%) who did not receive exclusive breastfeeding died (Table 4).

**Table 4:** Treatment and health service-related predictors of early neonates admitted to the neonatal intensive care unit of Bahir Dar City public hospitals, northwest Ethiopia, 2023.

Variable	Category	Total, *n* (%)	Status of the neonates	
			Censored, *n* (%)	Death, *n* (%)
ANC follow-up	Yes	378 (97.67)	322 (98.17)	56 (94.92)
	No	9 (2.33)	6 (1.83)	3 (5.36)
Number of ANC follow-up	1–3	173 (45.77)	139 (43.17)	34 (60.71)
	≥4	205 (54.23)	183 (56.83)	22 (39.29)
Time of breast-feeding initiation	Within 1 h	194 (50.13)	163 (49.70)	31 (52.54)
	More than 1 h	193 (49.87)	165 (50.30)	28 (47.46)
Mode of delivery	SVD	274 (70.80)	229 (69.82)	45 (76.27)
	C/S	113 (29.20)	99 (30.18)	14 (23.73)
Length of stay	≤1 day	43 (11.11)	23 (7.01)	20 (33.90)
	>1 day	344 (88.89)	305 (92.99)	39 (66.10)
PNC	Yes	324 (83.72)	284 (86.59)	40 (67.80)
	No	63 (16.28)	44 (13.41)	19 (32.20)
Trained NICU	Yes	265 (68.48)	222 (67.68)	43 (72.88)
	No	122 (31.52)	106 (32.32)	16 (27.12)
Resuscitation	Yes	60 (15.50)	16 (4.88)	44 (74.58)
	No	327 (84.50)	312 (95.12)	15 (25.42)
KMC given	Yes	26 (6.72)	24 (7.32)	2 (3.39)
	No	361 (93.28)	92.68 (92.68)	57 (96.61)
Birth attendant	Health professional	373 (96.38)	9 (2.74)	54 (91.53)
	TBA/family	14 (3.62)	318 (96.95)	5 (8.47)
Place of delivery	Health facility	373 (96.38)	318 (96.95)	55 (93.22)
	Home	14 (3.62)	10 (3.05)	4 (6.78)
Exclusive breast feed	Yes	269 (69.51)	247 (75.30)	22 (37.29)
	No	118 (30.49)	81 (24.70)	37 (62.71)

### New medical problems developed during the follow-up

**Figure 1:**
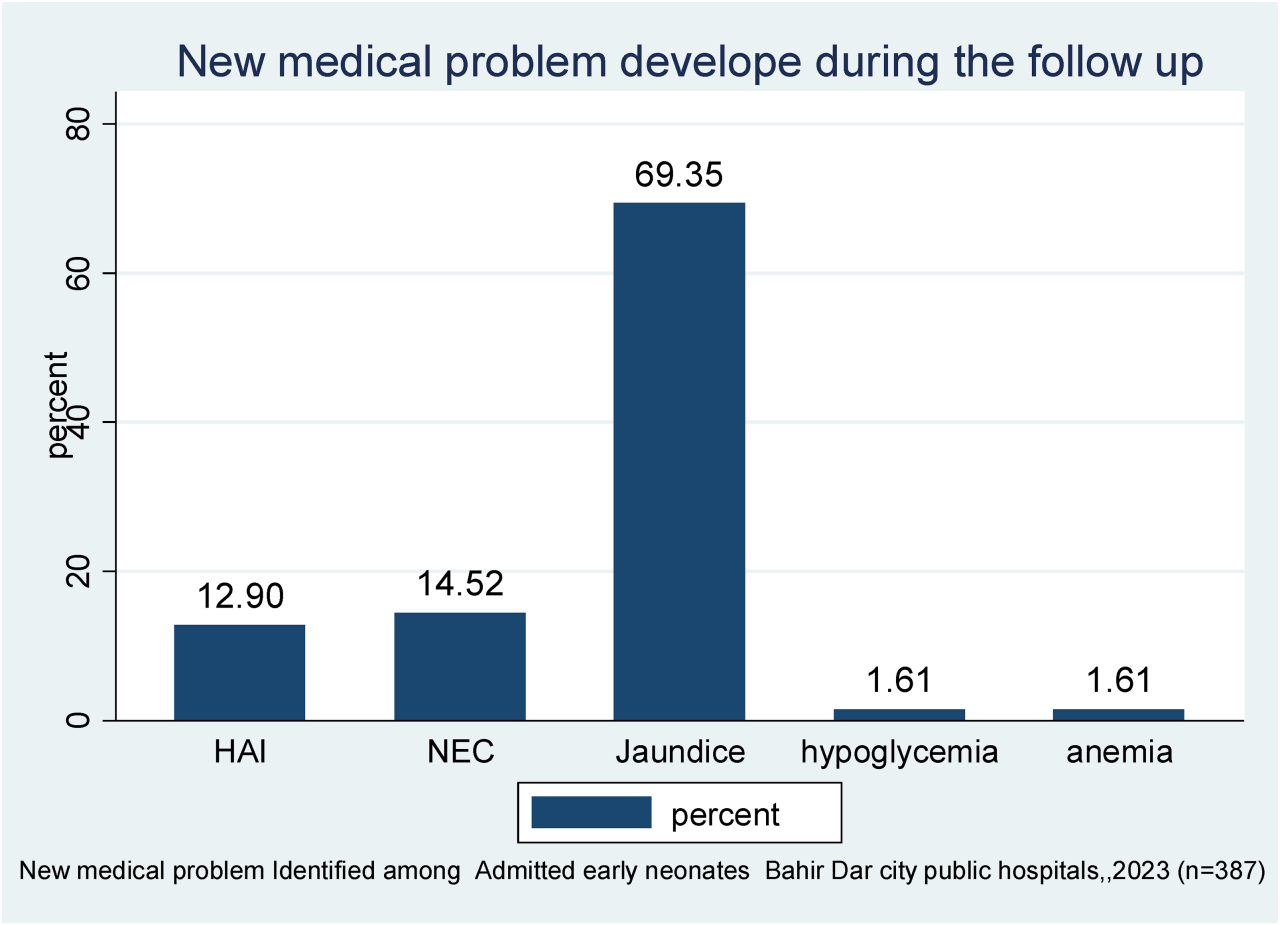
New medical problems identified during the follow-up period of early neonates admitted to the neonatal intensive care unit of Bahir Dar City public hospitals, northwest Ethiopia, 2023 (*n* = 387).

Of 387 followed early neonates, 62 (1.60%) developed new medical problems. Among those, 43 (69.35%) developed jaundice, followed by NEC (14.52%) and hospital-acquired infection (HAI) (12.9%). In total, 11 (68.75%) early neonatal deaths were observed in those who had developed jaundice (Figure 1).

### Outcome of admitted early neonates

**Figure 2:**
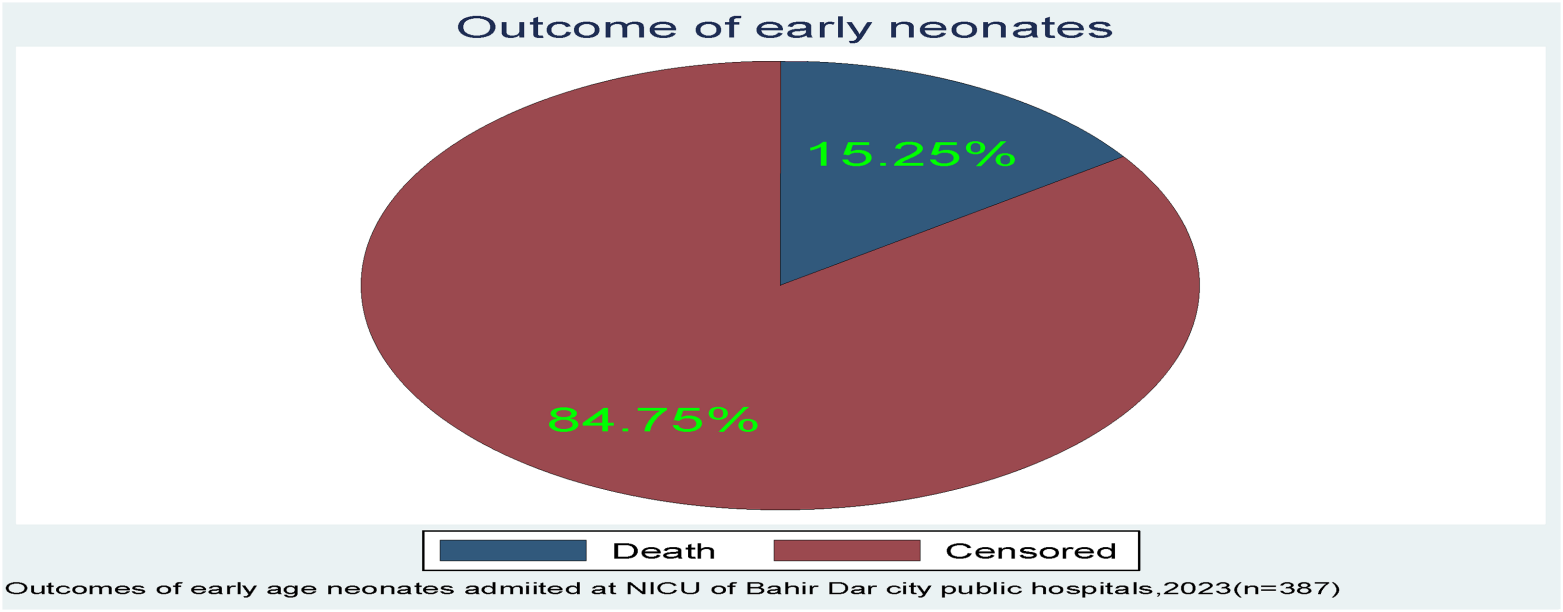
The outcome of early neonates admitted to the neonatal intensive care unit of Bahir Dar City public hospitals, northwest Ethiopia.

A total of 387 early neonates were followed for up to 7 days, beginning at the time of admission and continuing until the age of 7 days or the occurrence of an event. However, the neonate stayed for a range of 0.17–7 days. Among those early neonates, the incidence of death was 15.25% (*n*=59; 95% CI: 0.12–0.19) (Figure 2).

### Causes of early neonatal death

**Figure 3:**
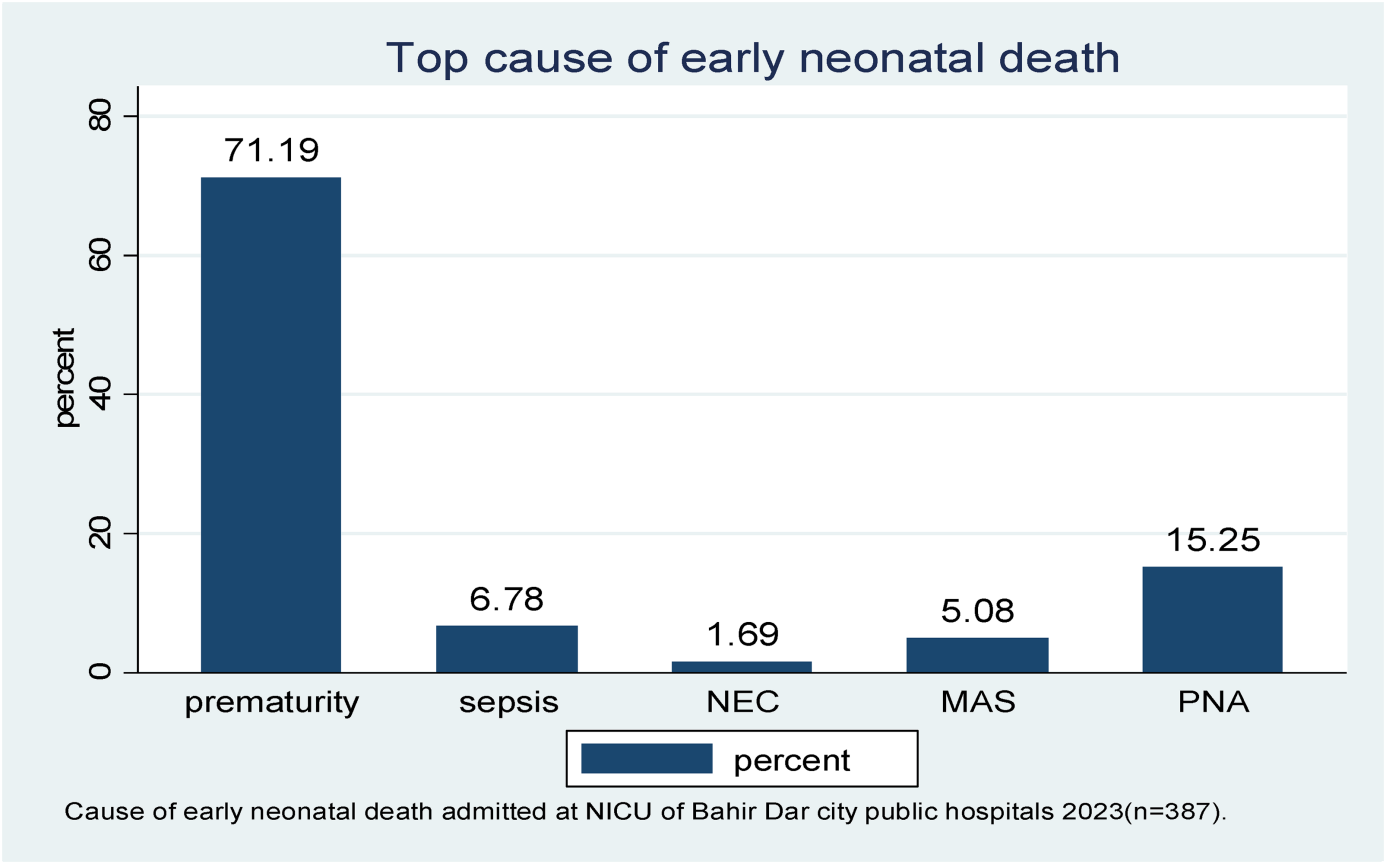
The leading cause of early neonatal death admitted to the neonatal intensive care unit of Bahir Dar City public hospitals, northwest Ethiopia, 2023 (*n* = 387). *NEC, necrotizing enterocolitis; MAS, meconium aspiration syndrome; PNA, perinatal birth asphyxia.

In this follow-up study, the major causes of early neonatal death were prematurity-related complications, birth asphyxia, and early neonatal sepsis (71.19%, 15.25%, and 6.78%, respectively) (Figure 3).

**Figure 4:**
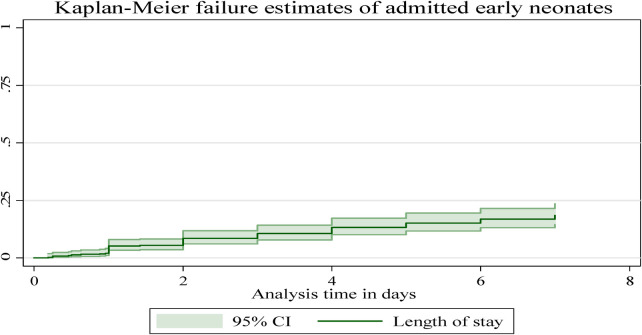
The overall Kaplan–Meier failure estimates of early neonatal deaths admitted to Bahir Dar City public hospitals, northwest Ethiopia, 2023.

In this follow-up study, the median time to early neonatal death was not attained; as explained in Figure 4, after admission, the graph started to increase, indicating a higher probability of a hazard of early neonatal death, and in the remaining follow-up days, the hazard of death rose as the length of hospital stay increased (Figure 4).

### Failure/death of the early neonates

In this follow-up study, the neonates stayed for different periods, making the entire observation 1,855.53 neonate days. As mentioned above, the overall incidence of death in the follow-up was 15.25% (*n*=59; 95% CI: 0.12–0.19), and the overall incidence density rate in this study was 31.79 per 1,000 early neonatal days (95% CI: 0.024–0.041). The incidence across different categories was different. As an example, the incidence of early neonates with RDS and those without RDS was 144.42 per 1,000 early neonate days (95% CI: 103.19–202.12). This study revealed that 33.90% (95% CI: 0.23–0.47), 33.90% (95% CI: 0.23–0.47), and 32.20% (95% CI: 0.21–0.45) of early neonatal deaths occurred in the first 24, 24–72 h, and 4–7 days, respectively. The median survival time was not attained because more than 78% of the early neonates survived beyond the study time; rather, we calculated the cumulative and mean survival times. The mean (±SD) survival time to early neonatal death was 2.72 ± 1.96 days, with a minimum and maximum length of stay of 0.08 and 7 days, respectively. This study showed that the cumulative failure probability was 2.08% (95% CI: 0.01–0.04) and 21.60% (95% CI: 0.17–0.28) at the end of day 1 and day 7, respectively, and as the length of stay in the hospital increased, the hazard of early neonatal death increased (Table 5).

**Table 5:** Life table estimation of time to early neonatal death admitted to the neonatal intensive care unit at the Bahir Dar City public hospitals, northwest Ethiopia, 2023.

Interval days	Beginning total	Event	Withdraw from study	Survive time	Cum. survive	Cum. Failure	Hazard rate
0–1	387	8	3	0.9792	0.9792	0.0208	0.0210
1–2	376	13	20	0.9445	0.9249	0.0555	0.0362
2–3	343	11	30	0.9128	0.8442	0.0872	0.0341
3–4	302	7	23	0.8908	0.7520	0.1092	0.0244
4–5	272	8	34	0.8629	0.6489	0.1371	0.0319
5–6	230	5	31	0.8427	0.5468	0.1573	0.0236
6–7	194	7	187	0.7840	0.4287	0.2160	

#### Comparison of time to early neonatal death for different categorical predictors

**Figure 5:**
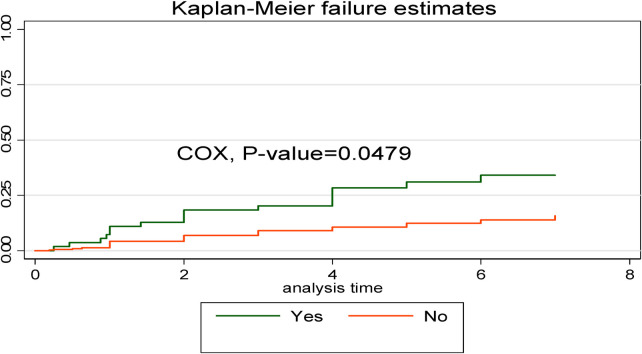
Kaplan–Meier curve with Cox equality test comparison of birth asphyxia for time, cause of early neonatal death admitted to the neonatal intensive care unit of Bahir Dar City public hospitals, northwest Ethiopia, 2023.

The Kaplan–Meier failure curve estimate with the Cox survival equality test for various covariates is illustrated in Figure 5. According to this study, the early neonates who were admitted with asphyxia had a higher risk of dying throughout their hospital stay than the early neonates who were not admitted with asphyxia. This emphasizes that as the length of hospital stay increased, early neonates who had been diagnosed with asphyxia had a shorter median time than those without asphyxia (Figure 5).

**Figure 6:**
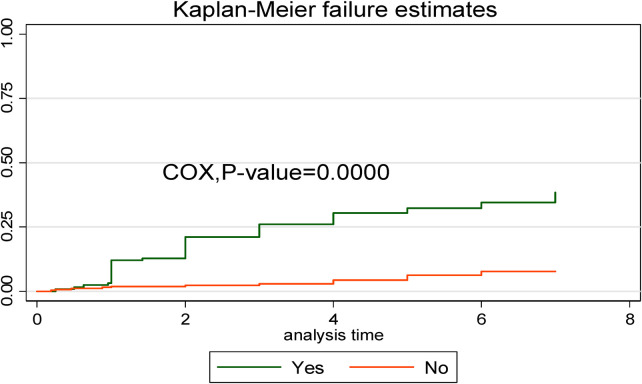
Kaplan–Meier curve with Cox equality test comparison of respiratory distress syndrome for time, causes of early neonatal death admitted to the neonatal intensive care unit of Bahir Dar City public hospitals, northwest Ethiopia, 2023.

In comparison to early neonates admitted without respiratory distress syndrome, early neonates with respiratory distress syndrome had a greater risk of dying. This showed that the time to early neonatal death was shorter in neonates with respiratory distress syndromes than in neonates without such syndromes (Figure 6).

In addition, a Kaplan–Meier estimate curve with a Cox regression-based test for equality of the survival function curve illustrates the impact of each covariate on early neonatal death (Table 6).

**Table 6:** Cox regression-based test for equality of survival curve for various categorical predictors of early neonatal death admitted to the neonatal intensive care unit at Bahir Dar City public hospitals northwest Ethiopia, 2023.

Variable	Categorical	Incidence rate/1,000 early neonates (95% CI)	*χ* ^2^	*p*-Value
Residence	Urban	23.43 (15.95, 34.41)	6.12	0.0133
	Rural	44.26 (31.46, 62.25)		
Occupation	Government employee	27.34 (13.04, 57.36)	12.99	0.0113
	Private	29.37 (14.69, 58.72)		
	Marchant	26.89 (8.67, 83.39)		
	Farmer	63.51 (41.82, 96.45)		
	Housewife	22.64 (14.44, 35.50)		
Gravidity	Primi gravida	23.39 (15.09, 36.26)	7.57	0.0227
	Multi gravida	37.56 (26.56, 53.11)		
	Grand-multigravida	47.14 (22.49, 98.89)		
Type pregnancy	Single	25.21 (18.18, 34.94)	7.28	0.0070
	Multiple	53.83 (35.77, 81.00)		
History of preterm	Yes	98.30 (49.16, 196.57)	8.01	0.0047
	No	28.75 (21.85, 37.82)		
Birth weight	<2,500 gm	49.88 (37.36, 66.59)	21.06	0.0000
	≥2,500	13.93 (8.09, 23.99)		
Size of GA	AGA	15.34 (9.66, 24.34)	10.92	0.0000
	SGA	70.32 (51.58, 95.86)		
	LGA	8.84 (1.25, 62.78)		
RDS	Yes	144.42 (103.19, 202.12)	58.10	0.0000
	No	15.43 (10.43, 22.84)		
Asphyxia	Yes	70.55 (43.86, 113.48)	3.91	0.0479
	No	26.01 (19.22, 35.20)		
Temperature	Yes	40.23 (30.40, 53.23)	8.51	0.0142
	No	15.69 (8.44, 29.15)		
5th min APGAR	<7	66.56 (35.81, 123.70)	12.15	0.0005
	≥7	19.53 (13.09, 29.13)		
Gestational age	<37 weeks	53.40 (39.60, 72.00)	21.87	0.0000
	≥37 weeks	15.23 (9.33, 24.87)		
Prematurity	Yes	53.40 (39.60, 72.00)	21.50	0.0000
	No	15.23 (9.33, 24,87)		
PNC	Yes	25.71 (18.86, 35.05)	11.31	0.0008
	No	63.40 (40.44, 99.40)		
EBF	Yes	16.37 (10.78, 24.86)	30.30	0.0000
	No	72.33 (52.41, 99.83)		
Resuscitation	Yes	221.83 (165.08, 298.09)	125.14	0.0000
	No	9.05 (5.46, 15.01)		
CPAP	Yes	103.31 (79.32, 134.56)	118.65	0.0000
	No	3.02 (1.13, 8.05)		

#### Predictors of time to early neonatal death

The Cox proportional hazard regression model was used to determine the predictors of time to early neonatal death. After a bivariable with a *p*-value <0.25, this included residence, sex, gravidity, type of pregnancy, history of preterm, admission weight, RDS, asphyxia, 5-min APGAR, PNC, complications during labor, admission temperature, size of gestational age, exclusive breastfeeding were candidates for multivariable Cox proportional hazard analysis.

In the multivariable proportional hazard model, gravidity, asphyxia, respiratory distress syndrome, size of gestational age, and exclusive breastfeeding were significantly associated predictors of time to early neonatal death at the NICU of Bahir Dar City public hospitals. After a multivariable Cox proportional hazard analysis, early neonates delivered from index mothers of multigravida and grand multigravida had a hazard of death 4 (AHR: 4.34; 95% CI: 1.63–11.55) and 3 (AHR: 3.50; 95% CI: 1.12–10.95) times higher compared to early-age neonates delivered from primigravida mothers, respectively. Early-age neonates who had been admitted with respiratory distress syndrome had a 2.60 (AHR: 2.60; 95% CI: 1.03–6.58) greater hazard of dying than those early-age neonates who had been admitted without respiratory distress syndrome. An early neonate who had been admitted with asphyxia had a sevenfold (AHR: 7.51; 95% CI: 2.30–24.51) greater hazard of dying than an early-age neonate who had been admitted without asphyxia. An early-age neonate who was small gestational age had a 2.05 times (AHR: 2.05; 95% CI: 1.08–4.92) increase in the hazard of death compared to those early-age neonates who were appropriate gestational age. An early-age neonate who did not start initiating exclusive breastfeeding had a threefold (AHR: 3.46; 95% CI: 1.52–7.88) increased hazard of death compared to those early-age neonates who did receive exclusive breastfeeding (Table 7).

**Table 7:** Bivariable and multivariable Cox proportional hazard result of time, cause of early neonatal death admitted to the neonatal intensive care unit at Bahir Dar City public hospital, northwest Ethiopia, 2023.

Variable	Category	Status		CHR (95% CI)	AHR (95% CI)	*p*-Value
		Censored	Death			
Residence	Urban	202	26	1	1	
	Rural	126	33	1.91 (1.14, 3.19)	0.83 (0.37, 1.86)	0.653
Sex	Female	197	30	1	1	
	Male	131	29	1.41 (0.85, 2.35)	0.69 (0.33, 1.48)	0.346
Gravidity	Primigravida	143	13	1	1	1
	Multigravida	118	31	2.82 (1.47, 5.39)	4.34 (1.63,11.55)	0.003*
	Grand-multi gravida	67	15	2.36 (1.12, 4.97)	3.50 (1.12, 10.95)	0.032*
Tpregnancy	Single	257	36	1	1	
	Multiple	71	23	2.11 (1.25, 3.57)	0.99 (0.39, 2.55)	0.986
Hpreterm	Yes	9	8	3.56 (2.00, 6.32)	3.40 (0.95, 12.14)	0.060
	No	319	51	1	1	
Admission weight	<2,500 g	148	46	3.56 (1.92, 6.58)	0.40 (0.10, 1.57)	0.190
	≥2,500 g	180	13	1	1	
RDS	Yes	25	34	8.85 (5.27,14.86)	2.60 (1.03, 6.58)	0.044*
	No	303	25	1	1	
Asphyxia	Yes	38	17	2.62 (1.49, 4.61)	7.51 (2.30, 24.51)	0.001*
	No	290	42	1	1	
5th min APGAR score	<7	23	10	3.08 (1.47, 6.44)	0.68 (0.19, 2.45)	0.556
	≥7	210	24	1	1	
PNC	Yes	284	40	1	1	0.053
	No	44	19	2.50 (1.45, 4.32)	2.45 (0.99, 6.06)	
Comp. labor	Yes	98	14	0.68 (0.38, 1.25)	1.45 (0.63, 3.33)	0.385
	No	230	45	1	1	
Hypothermia	Yes	198	49	2.65 (1.34, 5.24)	0.88 (0.35, 2.22)	0.787
	No	130	10	1	1	
Size for GA	AGA	225	18	1	1	1
	SGA	80	40	4.21 (3.12, 7.30)	2.05 (1.08, 4.92)	0.038*
	LGA	23	1	2.12 (1.017, 6.88)	0.30 (0.02, 4.07)	0.368
		23	1			
EBF	Yes	247	22	4.29 (2.53, 7.27)	3.46 (1.52, 7.88)	0.003*
	No	81	37	1	1	1

CHR, crud hazard ratio; AHR, adjusted hazard ratio; SGA, small for gestational age; AGA, appropriate for gestational age; LGA, large for gestational age; Tpregnancy, type of pregnancy; Comp. labor, complication during labor; Hpreterm, history of preterm.

*Significant at *p* < 0.05.

## Discussion

The aim of this study was to determine the time, causes, and predictors of ENDs in Bahir Dar City public hospitals in northwest Ethiopia. The leading causes of early neonatal death were prematurity-related complications, perinatal birth asphyxia, sepsis, meconium aspiration syndrome, and necrotizing enterocolitis. The overall incidence of death and mean time to death were 15.25% and 2.72 days, respectively. Gravidity, RDS, birth asphyxia, small gestational age, and being unable to initiate exclusive breastfeeding were independent predictors of time to early neonatal death.

In this study, the leading causes of early neonatal death were prematurity-related complications, birth asphyxia, and early neonatal sepsis, respectively, and 33.9%, 67.8%, and 32.2% of early neonatal deaths occurred in the first 24, 72 h, and 4–7 days, respectively. This was supported by studies conducted in India, Eritrea, and Dessie (21, 36, 37). This might be because most of the early neonatal deaths in resource-limited countries such as Ethiopia are associated with preterm delivery, intrapartum, newborn care practices, low birth weight, not receiving ANC, small gestational age, low APGAR score, delayed start of breastfeeding, home birth, and hypoxia (19–24).

Overall, in this study, 59 (15.25%) early neonates died, which makes the early neonatal mortality rate 152.5 per 1,000 live births. This study is in line with those carried out at Mekelle General and Ayder Compressive Specialized Hospitals (20) and Ethio-Somali (22). In this study the incidence of early neonatal death was also higher than the study conducted at Dessie (21) and Debre-Markos (15). The possible explanation for this difference might be study design, availability of medical equipment, NICU setup, sample size difference, and the fact that nearly all of the critically ill neonates in our study area were brought in from referred cases throughout the Amhara region, including Benishangul Gumuz region. The numbers in our study were also higher than the national-level population-based survey (41.8 deaths per 1,000 live births) (38) and the average national mini-EDHS 2019 (33 deaths per 1,000 live births). A possible explanation might be that because our study was conducted in the NICU, those critical neonates were admitted while the national average mini-EDHS included high-risk, low-risk, full neonatal period, and healthy neonates. This made the numbers in our study higher than average. The numbers in this study were also higher than those in studies conducted in Austria, Chicago, China, and Iran (39–42). This discrepancy might be due to a sample size difference, accessibility of health service utilization, poor ANC coverage, socioeconomic differences, and NICU setup differences. On the contrary, the numbers in this study were lower than those in a study conducted in Eritrea (37). A possible explanation might be that the study area in Eritrea was highly populated, more fertile, and conducive to settlement, and there was one subzonal hospital that caused high patient flow and might cause high numbers of ENDs (37). Other explanations might be study design differences, sample size, accessibility of NICU setup, socioeconomic factors, and inadequate distribution of health professionals.

This study revealed that the overall incidence density rate was 31.79 per 1,000 early neonate days (95% CI: 0.024–0.041), which was higher than the study conducted in a systematic meta-analysis of SSA, which was 22.51 per neonate day. This discrepancy might be because this study was conducted in an NICU, where early neonates required urgent critical care, whereas the study conducted in SSA covered a large geographical area, used secondary data, and included both the NICU and rural communities.

In this study, the median survival time was not attained since more than 78% of early neonates survived beyond the median time, which was similar to that in studies conducted in Northeast Amhara (21) and Tigray, northern Ethiopia (20). This similarity could be a shorter follow-up period and a greater proportion of right-censored. In our study, the mean survival time for END was 2.72 days. To the best of our knowledge, no other studies with a similar age group have reported their findings.

In the present study, early neonates who delivered from having multigravida and grand multigravida had a 4 (AHR: 4.34) and 3 (AHR: 3.50) times higher hazard of END compared to those early neonates delivered from primigravida mothers, respectively. This finding is supported by studies in China (41), Wollega (8), and southern Ethiopia (43). This may be due to multiparity-related obstetric complications that lead to bad outcomes, socioeconomic-related factors, or advanced maternal age-related physiological degeneration. In addition, as the mother's birth order increases, there might be a short birth interval, which may cause maternal depletion syndrome and resource competition between siblings (44).

The current study revealed that early neonates who had been admitted with RDS had a more than twofold (AHR = 2.60) hazard of END compared to those early neonates admitted without RDS. This is supported by studies conducted in Ghana (12), India (4), northeast Amhara, Dessie (21, 45), and Debre-Markos (15). A possible explanation might be RDS, mostly related to prematurity: either surfactant deficiency or inactivation; poor ANC service utilization; low maternal body mass index; low birth weight; oligohydramnios; fetal distress; and birth asphyxia. All this predisposes the neonates to develop END (46, 47), and in our study, more than 71% of deaths were caused by prematurity-related complications; hence, the problem of lung immaturity is a common phenomenon that leads to lung collapse and respiratory failure (48).

This study showed that early neonates who had been admitted with birth asphyxia had a sevenfold (AHR 7.51) increased hazard of death compared to those early neonates who had been admitted without birth asphyxia. This study was similar to studies conducted in Ghana (12), Rwanda (49), southern Ethiopia (50), the Ethio-Somali region (51), northern Ethiopia (33, 52), and northeast Amhara (21, 45). A possible explanation might be fetal hypoxia, which impairs the exchange and transportation of oxygen from the mother to the fetus during delivery. Fetal hypoxia compromises the oxygen supply to the fetus’ essential organs, leading to irreparable organ damage and death (53).

Regarding this study, early neonates who had been admitted at a small gestational age had a twofold (AHR, 2.05) increase in the hazard of END compared to those early neonates admitted at an appropriate gestational age. This finding is similar those in studies conducted in Afghanistan (54), Nigeria (55), Pakistan (56), Jimma (57), Addis Ababa (13), and southern Ethiopia (58). A possible scientific explanation for this might be that at a small gestational age, early neonates could be at risk of developing low birth weight and might be born prematurely. Prematurity predisposes early neonates to a higher risk of infection, hypoglycemia, hypothermia, RDS, anemia, and newborn jaundice (59). In contrast, a Nigeria DHS (60) revealed that small neonates were less likely to die during the early neonatal period, while large neonates were more likely to die within 6 days. This might be large for gestational age neonates; early neonates are more likely to have macrosomia, which increases the risk of birth injuries, hypothermia, birth asphyxia, and MAS (61, 62).

In the present study, early neonates who were unable to start exclusive breastfeeding had a threefold (AHR: 3.46) higher hazard of dying than those who were able to start breastfeeding exclusively. This finding is supported by the study conducted in Arba Minch General Hospital (6), Tigray (33), Debre Markos (15), and Uganda (63). A possible explanation might be due to exclusive breastfeeding, especially the colostrum, which is rich in protective factors and reduces the occurrence of NEC, gastroenteritis, respiratory illness, sudden infant death syndrome, and hypoglycemia (64).

## Strength and limitation

### Strength

This study's fundamental strength is that it was conducted prospectively, which increases the quality of the data and covers important sociodemographic variables as well as all significant clinically plausible predictors. The study area also covers all public comprehensive hospitals, including the primary hospital in Bahir Dar City, which gives our study a high degree of representative power. The study's main limitation was the exclusion of early neonates who were delivered and died at home because the study was done in hospitals, and the outcomes of the referred and left-against were not known. There was END before initiating exclusive breastfeeding; this makes the true causal association between END and exclusive breastfeeding overvalued. Finally, we did not address the influence of institutional factors, such as equipment, supply, NICU nursing staff proportion, transportation details, care during transportation, and distance.

## Conclusion

The median survival time was not attained, the mean time to early neonatal death was 65 h, with a total analysis time at risk of 1,855.53 neonate-day observation, and the incidence of death was high compared to previous studies in Ethiopia. Most of the early neonatal deaths occurred during the first 72 h. The major causes of END were prematurity-related complications, birth asphyxia, sepsis, and MAS. The following statistically significant predictors were identified: early neonates delivered from multigravida and grand multigravida; birth asphyxia; RDS; SGA; and being unable to exclusively breastfeed. In the future, since this study found a high incidence of death compared to previous studies in Ethiopia and mini-EDHS 2019, researchers should carry out long-term prospective follow-up studies at a multicenter nationwide level.

## Data Availability

The original contributions presented in the study are included in the article/**Supplementary Material**, further inquiries can be directed to the corresponding author.
