# Supplementary material for: Time, cause of early neonatal death, and its predictors among neonates admitted to neonatal intensive care units at Bahir Dar City public hospitals, northwest Ethiopia: a prospective follow-up study
Source: Front Pediatr. 2024 Jun 11;12:1335858. doi: 10.3389/fped.2024.1335858 (PMC11196776; doi:10.3389/fped.2024.1335858)
Supplement: Supplementary file 1 [file Table1.pdf]

Supplementary Table 1:Sample size calculation for predictors of time, causes of early neonatal death among neonates admitted at Bahir Dar City public hospitals, Northwest Ethiopia, 2023.

Predictors	Hazard ratio	Stata calculation result	After adding 10%
Neonatal sepsis (21)	3.34<br>P1=0.090<br>P2=0.027	194	213
Low birth weight (21)	3.563<br>P1=0.096<br>P2=0.027	164	181
Respiratory distress syndrome (21)	4.018<br>P1=0.092<br>P2=0.023	143	158
Birth asphyxia (21)	2.54<br>P1=0.084<br>P2=0.033	351	**387**
Late breastfeed initiation (21)	4.094<br>P1=0.115<br>P2=0.028	112	124
Prematurity (20)	3.7<br>P1=0.107<br>P2=0.029	140	154

P1: the probability of the event in the exposed group, p2: the probability of the event in the unexposed group
