# Supplementary material for: Time, cause of early neonatal death, and its predictors among neonates admitted to neonatal intensive care units at Bahir Dar City public hospitals, northwest Ethiopia: a prospective follow-up study
Source: Front Pediatr. 2024 Jun 11;12:1335858. doi: 10.3389/fped.2024.1335858 (PMC11196776; doi:10.3389/fped.2024.1335858)
Supplement: Supplementary file 2 [file Table2.pdf]

Supplementary Table 2: Schoenfeld residual test result for Cox proportional hazard assumption for Time, causes of early neonatal death among neonates admitted to NICU Bahir Dar City public hospitals, Northwest, Ethiopia 2023.

Variable	Rho	Chi2	Df	Prob>Chi2
Residence	-0.01547	0.01	1	0.9103
Gravidity	0.30976	3.83	1	0.0504
History of preterm	0.01789	0.01	1	0.9036
Weight at admission	-0.07732	0.30	1	0.5840
ANC	-0.11692	0.57	1	0.4522
PNC	-0.05381	0.17	1	0.6819
Type of pregnancy	0.00385	0.00	1	0.9797
Complication of labor	0.12654	0.72	1	0.3962
Size of gestational age	-0.13557	1.26	1	0.2623
APGAR score	-0.10112	0.73	1	0.3935
Hypothermia	0.04388	0.06	1	0.8083
RDS	0.11808	0.49	1	0.4858
Asphyxia	0.01363	0.01	1	0.9040
EBF	-0.05631	0.14	1	0.7131
sex	-0.14356	0.91	1	0.3402
Occupation	0.05052	0.12	1	0.7274
Education	0.18256	1.62	1	0.2035
Place of delivery	0.00986	0.00	1	0.9492
**Global test**	15.37	18	**0.6366**	

*Rho is the correlation coefficient between residual and time
