# Supplementary material for: Time, cause of early neonatal death, and its predictors among neonates admitted to neonatal intensive care units at Bahir Dar City public hospitals, northwest Ethiopia: a prospective follow-up study
Source: Front Pediatr. 2024 Jun 11;12:1335858. doi: 10.3389/fped.2024.1335858 (PMC11196776; doi:10.3389/fped.2024.1335858)
Supplement: Supplementary file 3 [file Table3.pdf]

Supplementary Table 3:Multicollinearity Test

Multicollinearity test between the covariates of time, causes of early neonatal death, and its predictors among admitted at NICU of Bahir Dar City public hospitals, northwest, Ethiopia 2023(n=387).

Variable	VIF	1/VIF
Weight at admission	7.28	0.137313
Asphyxia	5.72	0.174689
APGAR score	5.08	0.196696
Size gestational age	4.71	0.212364
Complication of labor	3.53	0.283320
residence	2.84	0.351568
RDS	2.67	0.373876
EBF	2.60	0.384860
Gravidity	2.42	0.413583
sex	2.23	0.448813
History preterm	2.04	0.490011
Type of pregnancy	1.97	0.507223
Hypothermia	1.69	0.591675
PNC	1.65	0.605180
Mean VIF **3.32**		
