# Supplementary material for: Time, cause of early neonatal death, and its predictors among neonates admitted to neonatal intensive care units at Bahir Dar City public hospitals, northwest Ethiopia: a prospective follow-up study
Source: Front Pediatr. 2024 Jun 11;12:1335858. doi: 10.3389/fped.2024.1335858 (PMC11196776; doi:10.3389/fped.2024.1335858)
Supplement: Supplementary file 5 [file Image2.pdf]

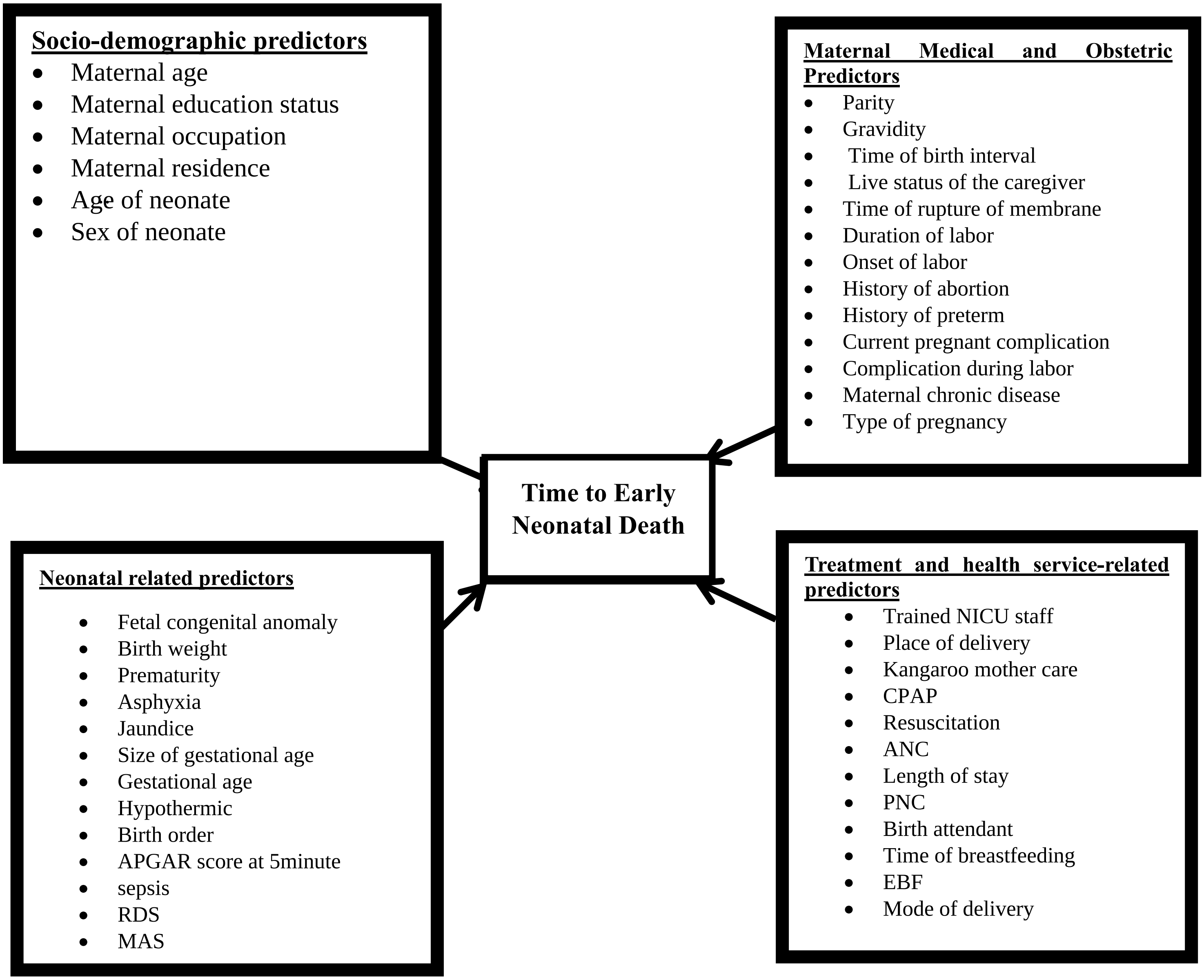

Supplementary Figure 2: Conceptual framework on time, causes of early neonatal death, and predictors among neonates admitted to neonatal intensive care units at Bahir Dar City public hospitals, Ethiopia 2023.
